# Bronchopulmonary Nematodes in Alpine Ibex: Shedding of First Stage Larvae Analyzed at the Individual Host Level

**DOI:** 10.3389/fvets.2021.663268

**Published:** 2021-04-29

**Authors:** Stefania Zanet, Ezio Ferroglio, Filippo Orlandini, Bruno Bassano, Elena Battisti, Alice Brambilla

**Affiliations:** ^1^Department Veterinary Sciences, University of Turin, Turin, Italy; ^2^Gran Paradiso National Park, Alpine Wildlife Research Center, Noasca, Italy; ^3^Department of Evolutionary Biology and Environmental Studies, University of Zurich, Zurich, Switzerland

**Keywords:** *Capra ibex*, lungworms, pneumonia, protostrongylidae, dictyocaulidae

## Abstract

Pneumonia is the most frequent cause of death for Alpine ibex (*Capra ibex*) in Gran Paradiso National Park, (Italy). The etiology of this form of pneumonia is currently unknown and the identification of the primary etiological agent remains difficult due to biological and logistic constraints. Uncovering individual differences in Protostrongylid prevalence and intensity is important to further investigate the epidemiology of respiratory diseases and their relationship to heterozygosity and inbreeding in a once almost extinct population like *C. ibex*. In a group of 21 individually recognizable adult male we monitored monthly prevalence and intensity of Protostrongylid first-stage larvae using Baerman's technique from June to September 2019. First-stage larvae of 5 genera were detected. *Muellerius* (*P* = 100%, CI_95%_ = 84–100) and *Protostrongylus* (*P* = 86%, CI_95%_:71–100) were two dominant genera according to Bush's importance index. *Neostrongylus* (*P* = 38%,CI_95%_: 17–59), *Cystocaulus* (*P* = 33%,CI_95%_ = 13–53) were classified as co-dominant genera while *Dictyocaulus filaria* (*P* = 0.05%, CI_95%_ = 0.04–0.13) was detected, for the first time in Alpine ibex, in one subject. Protostrongylidae larval excretion varied significantly over time, with minimum L1 excretion in July. Individual median larval intensity ranged from 4.4 lpg to 82.2 lpg with Poulin's discrepancy index showing highly aggregated distribution patterns for *Muellerius spp*. (*D* = 0.283, CI_95%_ = 0.760–0.895) and *Protostrongylus* spp. (*D* = 0.635, CI_95%_ = 0.580–0.705). Presented data provide the necessary base point to further investigate how lungworm infection account for the different rates of progression of pneumonia in *C. ibex*. Individual aggregation of larval intensity must be further evaluated to determine whether these differences mirror different levels of parasitic infection related to individual differences in immune response, hormonal-states or genetic fitness.

## Introduction

The Alpine ibex *Capra ibex* is a wild ungulate currently distributed across the European Alps in Italy, France, Switzerland, Lichtenstein, Austria, Germany and Slovenia (1). Despite being classified as a species of least concern in the International Union for Conservation of Nature's (IUCN) Red List of threatened species (2), *C. ibex* is currently object of multiple conservation efforts (Gruppo Stambecco Europa, Alpine ibex European Specialist Group -GSE-AIESG; Interreg-Alcotra 2014–2020 Lemed-Ibex). The recent history of *C. ibex* has been characterized by a strong bottleneck in which the Gran Paradiso colony of *C. ibex* (Gran Paradiso National Park – GPNP, north-western Italian Alps) was the only remnant population from which all conservation and reintroduction programs took origin (1). At the beginning of nineteenth century, it is estimated that no more than 100 individuals remained in the Gran Paradiso colony (1, 3). Low levels of heterozygosity, inbreeding, and inbreeding depression (4, 5), also found within the Gran Paradiso population, were shown to be associated with individual differences in fitness-related traits (6) and in disease susceptibility, particularly to infectious keratoconjunctivitis (7). One of the main concerns for the conservation of the species, which can be related to the above-mentioned genetic issues (7) arose from the observation of several epidemic outbreaks of disease. Examples are: sarcoptic mange in the eastern Alps (8), brucellosis in the Bargy Massif, France (9), infectious kerato-conjunctivitis in Gran Paradiso (7) and in Switzerland (10) and respiratory diseases in the Vanoise National Park, France (11). Moreover, in the last years several cases of mortality due to respiratory diseases were observed also in other areas of the Alps (GPNP, unpublished data). As the etiology of current respiratory disease affecting Alpine ibex remains unknown, insights on it appear essential.

Bronchopulmonary nematodes of the Families Protostrongylidae and Dictyocaulidae are commonly found to infect both domestic and wild ruminants (12). In wild ungulates, infections are commonly asymptomatic (12, 13). However, depending on the severity of infection, age, immunological status of the animal and predisposing factors, clinical signs of verminous bronchopneumonia may range from moderate coughing with increased respiratory rates to severe coughing, persistent respiratory distress and failure (14). Primary verminous pneumonia is seldom cause of death in wild ungulates (15, 16) while secondary bacterial infections are major cause of fatal respiratory disease (17). Several studies have assessed the respiratory helmintofauna of *C. ibex*, as well as seasonal patterns of infection at population level (13, 18, 19). However, susceptibility to disease can vary between and within populations because of differences in the environment but also because of individual characteristics such as age, sex and genetic traits (20, 21). Understanding whether there are individual differences in the susceptibility to bronchopulmonary nematodes infection, would thus be important to further investigate the epidemiology of respiratory diseases and their relationship to heterozygosity and inbreeding. In this study, we assessed the larval excretion on bronchopulmonary nematodes larvae on individually recognizable male ibex in order to identify endogenous and exogenous variables affecting the degree larval excretion.

## Materials and Methods

### Sample Collection and Analysis

Twenty-one male Alpine ibex were sampled monthly from June to September 2019 in GPNP area of Levionaz (Valsavarenche, Italy) at elevation varying from 1,500 m a.s.l. in the month of June, to 2,900 m a.s.l. in the month of August. Sampled subjects had been previously marked with ear-tags within a long-term research project for Alpine ibex conservation (Ecology and Conservation of the Alpine Ibex – Gran Paradiso National Park, Italy) and were therefore individually recognizable. Individuals included in the study were aged from 5 to 11 years old (mean = 8.19, sd = 1.69, Table 1).

**Table 1:** Sample distribution by age.

**Age**	**Number of ibex sampled**
5 years old	2
6 years old	2
7 years old	3
8 years old	3
9 years old	6
10 years old	4
11 years old	1

Fresh fecal samples were collected from each animal, by direct observation, every 30 days (±2 days) and conserved at +4°C, for a maximum period of 48 h, until further analysis. For more details on fecal collection see Brambilla et al. (22). Ten grams of faces were analyzed by Baermann technique (23). First-stage larvae (L1) of bronchopulmonary nematodes were detected and counted, using a light microscope (× 10 or × 40 magnification). The total number of larvae was divided by 10 to obtain the number of larvae per gram (*lpg*) of feces. L1 of bronchopulmonary nematodes were morphologically identified using appropriate identification keys (24). Where necessary, the identity of L1 was confirmed by PCR targeting a specific fragment of the ITS2 gene (25).

### Statistical Analysis

The statistical analyses were carried out with R version 3.5.2 software (26).

Epidemiological characteristics including prevalence (overall and monthly percentage of infected host individuals) and intensity of infection (overall and monthly mean number of parasites per infected host) were calculated for each parasite.

To evaluate the effect of month and age on parasite distribution, we built a generalized linear model with negative binomial distribution (package glmmTMB) (27). The fixed part of the model included month and age as explanatory variables and the total number of larvae as dependent variable. Pairwise comparison of different months was performed with emmeans package (28) that compute estimated marginal means for specified factors. The effect of individual identity on larval excretion was tested by AIC comparison (29) of two models having the same fixed structure as described above. The first model also included the individual identity as a random term (mixed effect model) while in the second model, the random term was omitted. Importance value suggested by Bush (I) was used to characterize the importance of each genera in the parasite community (30, 31). Additionally, for the genera detected at all sampling times, the Poulin's discrepancy index (D) was assessed (32, 33).

## Results

First-stage larvae of 5 genera were detected, namely *Muellerius, Protostrongylus, Cystocaulus, Neostrongylus*, and *Dictyocaulus*. The overall prevalence of infection was 100% since, in all subjects, L1 were detected at least once during the study.

Prevalence of infection varied greatly among genera. Over the whole study period, the most prevalent parasite was *Muellerius* spp. found in 21/21 ibex (*P* = 100%, CI_95%_= 84–100), followed by *Protostrongylus* spp. found in 18/21 ibex (*P* = 86%, CI_95%_:71–100). Moderate prevalence was detected for *Neostrongylus* spp. found in 8/21 ibex (*P* = 38%,CI_95%_: 17–59) and *Cystocaulus* spp. 7/21 ibex (*P* = 33%,CI_95%_= 13–53), whereas *Dictyocaulus* spp. was found only in one ibex (*P* = 0.05%,CI_95%_ = 0.04–0.13). Table 2 shows the total and monthly prevalence per each parasite genera.

**Table 2:** Total and monthly prevalence (CI95%) from June to September 2019, are reported for each of the 5 genera of lungworms detected by Baermann technique.

**Month**	***Muellerius* spp**.	***Protostrongylus* spp**.	***Neostrongylus* spp**.	***Cystocaulus* spp**.	***Dictyocaulus* spp**.
June	95% (86–100)	86% (71–100)	33% (13–53)	0	5% (4–13)
July	57%(36–78)	38%(17–59)	0	5% (4–14)	0
August	95% (86–100)	62%(41–83)	0	19%(7–40)	0
September	95% (86–100)	19%(22–36)	9%(3–22)	9%(3–22)	0
Total	100% (84–100)	86% (71–100)	38%(17–59)	33%(13–53)	(0.04–0.13)

*Muellerius* spp. was detected with constant prevalence (*P* = 95%, CI_95%_ = 86–100%) in June, August and September. The lowest prevalence was recorded in the month of July when only 57% of the tested animals were positive (CI_95%_ = 36–78%). Larvae of the genus *Protostrongylus spp*. were detected at each of the 4 sampling times with prevalence ranging from 86% (CI_95%_ = 71–100%) in June, to 19% (CI_95%_ = 22–36%) in September. Larvae of the genus *Cystocaulus spp*. were detected only from July to September with a peak prevalence of 19% (CI_95%_ = 7–40%) in August. L1 of *Neostrongylus spp*. and *Dictyocaulus spp*. were detected intermittently in June and September. The identity of *Dictyocaulus filaria* L1 was confirmed by means of PCR (97% homology to GenBank Accession number U37717.1 and registered in Genbank under Accession number MW057412).

The mean intensity over the whole study period were: 4.8 (sd = 6) for *Muellerius* spp., 3.6 (sd = 7) for *Protostrongylus* spp., 3 (sd = 7), for *Neostrongylus* spp. and 0.77 (sd = 0.4) for *Cystocaulus* spp. It was not possible to compute the intensity for *Dictyocaulus* spp. as it was detected only in one individual (0.2 lpg) in June.

The number of excreted L1 of all 5 detected genera, varied greatly among individuals, ranging from 4.4 to 82.2 lpg (mean 25.2). No differences were recorded based on animal's age (*p* > 0.05).

The monthly mean shedding intensity (and standard deviation, sd) for each of the detected genera is reported in Table 3. *Muellerius* spp. and *Protostrongylus* spp. were two genera detected at all sampling times. In July, intensity was not computed for *Cystocaulus* spp. as it was detected only in one ibex with 0.4 lpg.

**Table 3:** Monthly mean Intensity (mean number of L1/g calculated for the total of infected animals) and the relative standard deviation (sd) for each parasite is reported together with the monthly mean intensity for all detected species.

**Month**	***Muellerius* (sd)**	***Protostrongylus* (sd)**	***Neostrongylus* (sd)**	***Cystocaulus* (sd)**	***Dictyocaulus* (sd)**	**Mean Intensity (sd)**
June	9 (8.4)	7 (10)	3.8 (8.2)	NA	NA	15.7 (17.7)
July	1.3 (1.2)	0.5 (0.5)	NA	NA	NA	1.4 (1.2)
August	2.2 (2.7)	1.3 (1.8)	NA	0.7 (0.4)	NA	3.2 (4)
September	6.1 (5.3)	1.1 (1.1)	0.2 (0)	1.1 (0.1)	NA	6.5 (5.4)

**Figure 1:**
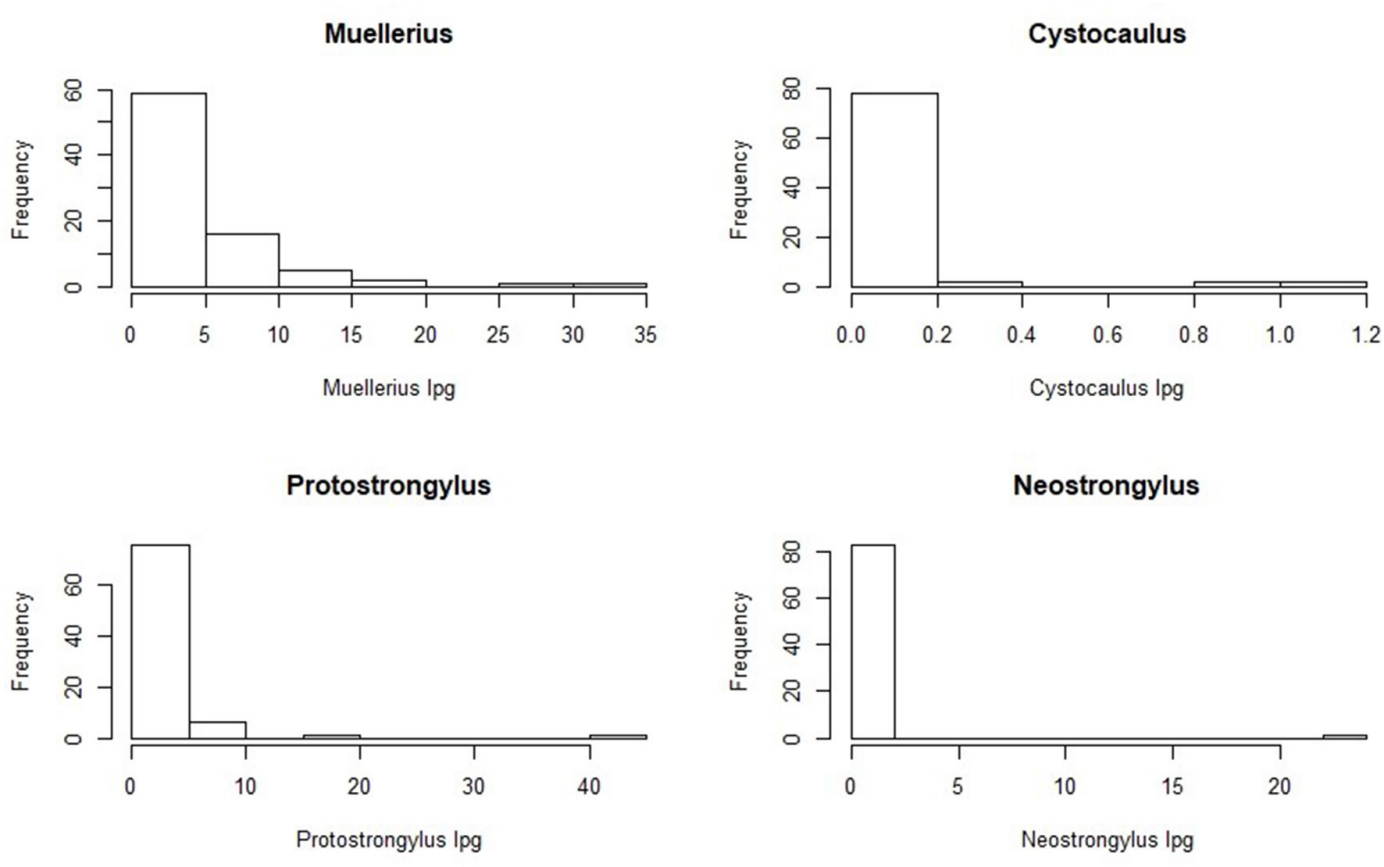
Median lpg values of broncho-pulmonary nematodes are represented in relation to month of sampling.

Regarding the effect of month on total larval shedding, the models showed that there was a significant variation of intensity over time (Figure 1).

The pairwise comparison between months showed that the monthly lpg values were significantly lower in July and August compared to June, and in July compared to September (Table 4).

**Table 4:** P-values of *post-hoc* test among monthly median values of lpg are reported.

	**June**	**July**	**August**
July	<0.001^*^	-	-
August	0.004^*^	0.144	-
September	0.134	0.005^*^	0.390

* *significant, < 0.05*.

**Figure 2:**
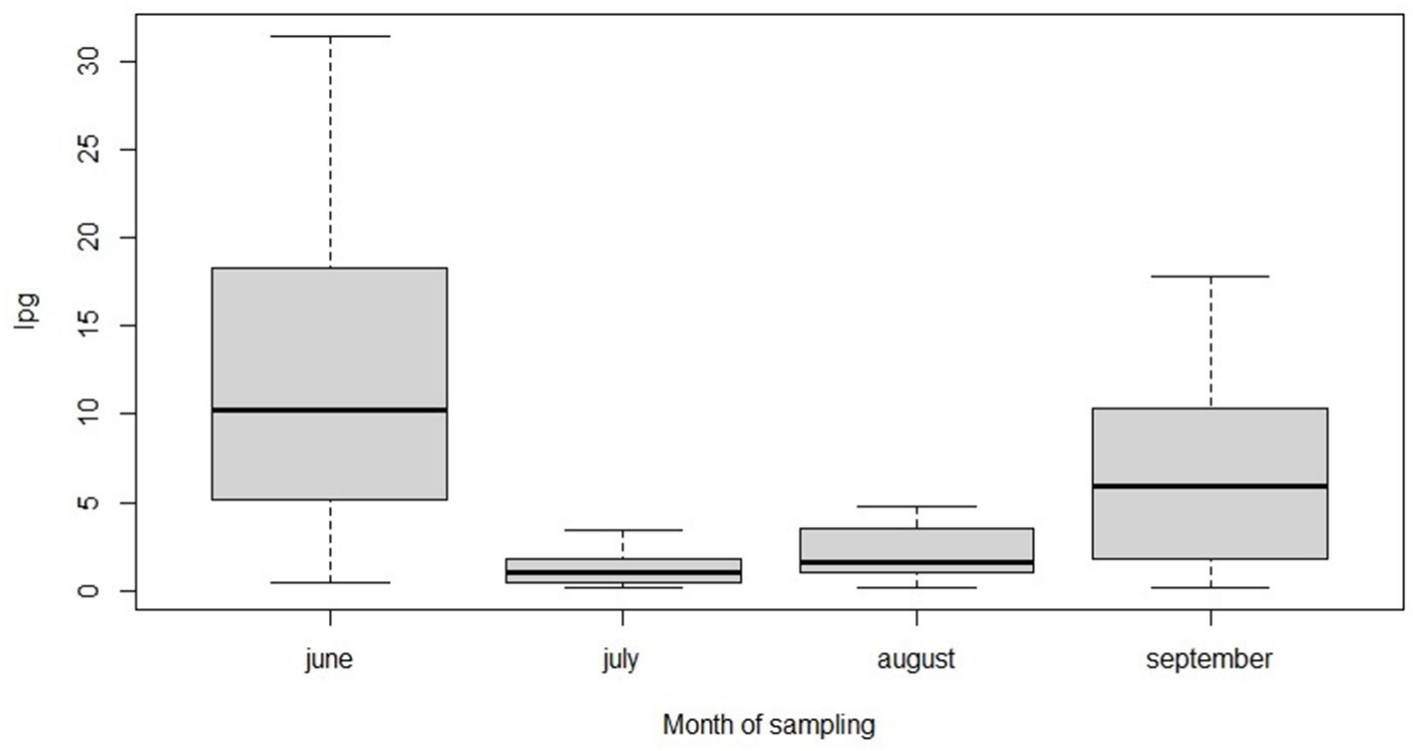
Seasonal median values of larval excretion (lpg) for each individual of the study. Lpg was assessed in May, June, July, August for each individual.

Age of the host did not have an effect on total larval shedding (*p* = 0.238). The comparison between the models including or omitting the individual identity as a random term did not allow to select one of the two models (Δ AIC = 1.3) (29, 34). A graphical representation of the larval excretion of the individuals is presented in Figure 2.

According to Bush's importance value, the structure of the community of bronchopulmonary nematodes exhibited 2 dominant genera, 2 co-dominant and one subordinate genus (Table 5).

**Table 5:** Classification and Bush's Importance values (I) for the bronchopulmonary nematodes of the Gran Paradiso Ibex colony.

**Dominant genera**	**I**
*Muellerius* spp.	79
*Protostrongylus* spp.	20
**Co-dominant genera**	
*Cystocaulus* spp.	0.12
*Neostrongylus* spp.	0.78
**Subordinate genera**	
*Dictyocaulus* spp.	0.0006

**Figure 3:**
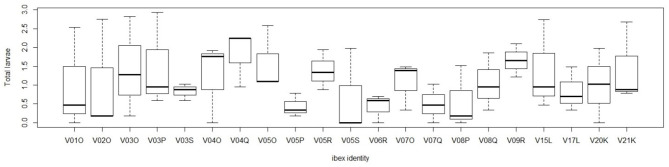
Frequency distribution of shedding intensity values for L1 of the 4 genera of Protostrongylidae.

The Poulin's discrepancy index (D) was 0.283 for *Muellerius spp*. (CI_95%_ = 0.760–0.895) and 0.635 for *Protostrongylus* spp. (CI_95%_ = 0.580–0.705). The frequency distribution of Protostrongylid nematodes is shown in Figure 3. The graphs show the aggregate distribution of L1 shedding, in which most samples are characterized by low L1 counts while only few individuals are highly parasitized.

## Discussion

Bronchopulmonary nematodes are a common finding in free-ranging wild ungulates (17, 35). Fecal excretion of first-stage larvae of bronchopulmonary nematodes has been used as valuable tool for a non-invasive assessment of parasite load and host–parasite relationships in wild ungulate populations (36). First-stage larvae of bronchopulmonary nematodes were detected in all 21 individuals (*P* = 100%) with a mean of 2.64 species (sd = 0.99) infecting each individual. A total of 5 genera were detected in the study, namely *Muellerius, Protostrongylus, Neostrongylus, Cystocaulus*, and *Dictyocaulus*. A previous study from Gran Paradiso National Park (15), conducted by identification of adult parasites in animals found dead, reported a prevalence of infection with three species of *Protostrongylidae*, namely *P. rufescens* (31.9%), *P. hobmaieri* (21.2%) and *Spicolocaulus austriacus* (46.8%). L1 of *S. austriacus*. are morphologically indistinguishable from those of *Protostrongylus* spp. (13, 24, 37). We must then assume that if present, L1 of *S. austriacus* in our study, were indeed accounted for as *Protostrongylus* spp. Interestingly, *Muellerius* spp. which is the species we reported with highest prevalence in this study and also the most common lungworm of sheep and goats in Europe (12), was not reported by Balbo et al. (15). Being *Muellerius* spp. a temperate Protostrongylid with lower tolerance to freezing temperatures (38), we can hypothesize that the increasing prevalence of this species can be related to progressively warming temperatures which allowed for a progressive upslope shift into alpine environments. This hypothesis is supported by recent studies in which *C. ibex* is found infested with the same four genera of Protostrongylidae encountered in Gran Paradiso National Park. Specifically, in four Ibex colonies in the Eastern Italian Alps, *Muellerius* spp. and *Protostrongylus* spp. were detected with a prevalence of 78.8 and 9.7%, respectively, while *Neostrongylus* spp. and *Cystocaulus* spp. were detected with a prevalence of 4.1 and 0.7% (13). Marreros et al. (18) investigated the prevalence of parasites in fecal samples of Alpine ibex in Switzerland between 2006 and 2008 and reported prevalence of infection varying from 79.9% for *Muellerius* spp. to 3.1% for *Cystocaulus* spp.

In the present study *Muellerius* spp. L1 were detected with a prevalence of 52. 6%, *Protostrongylus* spp. 15.8%, and *Neostrongylus* spp. 5.3%. *Muellerius* and *Protostrongylus* are the two genera that mainly characterize the parasitic community of *C. ibex* in the study area in GPNP as both were classified by Bush's Importance index as dominant genera. Bush's Importance index classified *Cystocaulus* and *Neostrongylus* as co-dominant genera as they were detected with lower prevalence and were not continuously detected over the 4 sampling times.

To our knowledge, this is the first report of *Dictyocaulus filaria* in Alpine ibex. *D. filaria* commonly infects both livestock (sheep and goat) (39) and wild ruminants (40, 41). In the same area of the Gran Paradiso National Park, a previous study by Balbo et al. (15) in which the authors examined the trachea, bronchi and lungs of 13 ibex and 71 chamois, only a single chamois was found infected with *D. filaria* while no data are available on this parasite's presence in sympatric livestock. Livestock is considered the main source of pasture contamination with *Dictyocaulus* spp. L1 and thus the main driver of infection (42). Although sampled animals do not simultaneously share pastures with livestock, portions of wintering areas of sampled ibex are used in summer by livestock and can be accounted as a possible source of mutual infection. Considered the single finding of *D. filaria*, the lack of previous reports in *C. ibex* as well as the single report of infection in chamois from GPNP, we confirm the Dictyocaulidae are marginal components of Ibex parasitic fauna in the Alps and possibly originating as occasional a spill-over from grazing livestock. On the contrary, Protostrongylids which ubiquitously infect *C. ibex* and are eliminated with highest intensity in spring (19), might be of concern for grazing livestock especially for lamb and calves at the beginning of grazing season.

Larval output intensity showed a well-defined temporal pattern across the study period. According to previous references, fecal excretion of broncho-pulmonary nematodes L1 reached its minimum in during summer (13, 43, 44) and in the case of the studied Gran Paradiso colony, intensity of L1 was minimal in July. Compared to data reported in previous studies, August was the month with minimal lungworm excretion in Swiss ibex colonies (18) and in the north-eastern Italian Alps (13). Due to the peculiar location of studied animals (high elevation with early autumn snow cover and late spring snow melting), the sampling was carried out only during the central months of summer and thus the complete seasonal variation of L1 excretion could not be assessed. In Lanfranchi et al. (19), where an annual assessment of L1 infestation was carried out on hunted animals, Protostrongylidae L1 peaked in April, month that corresponds to the end of the winter season and therefore to a time of considerable metabolic effort for the animals. *Cystocaulus* L1 were recorded only in the months of August and September, contrary to what reported by Marreros et al. (18) which instead detected *Cystocaulus* in the earlier part of summer from May to July. Intensity of larval excretion is lower in GPNP than in previously analyzed *C. ibex* populations (13, 18). In both cases the study design and methodology substantially differed from the one used in the present study, and comparison would be neither reliable nor informative. As suggested for Dall's sheep *Ovis dalli*, low larval output during summer, could reflect a seasonal decrease in parasite reproduction, or newly acquired infections which are not yet patent (35). However, the authors suggested that low larval output can also occur in animals with concurrent bacterial pneumonia due to inflammatory destruction and mechanical trapping of larvae in the airways (35).

Bush's Discrepancy Index, which measures aggregation of parasites within a population, confirms the highly aggregated distribution of investigated nematodes. *Protostrongylus* and *Muellerius* were detected at all sampling times and the first showed a highly aggregated distribution (*D* = 0.635), while *Muellerius* showed a less aggregated pattern (*D* = 0.283).

Despite it was not possible to select between the model including or excluding the individual identity, the larval excretion, calculated by adding together all the 5 genera of parasites, was characterized by high individual variation as it is also possible to observe from Figure 2. Individual median lpg values ranged from 4.4 to 82.2 lpg (mean 25.2). This wide variation of L1 excretion among sampled animals could not be explained by apparent individual characteristics such as age or sex, as all animals, belonged to the same colony and were males. Furthermore, no relationship was found between age and larval excretion. Individual differences in larval excretion mirror the level of parasitic infection of the host but might also depend from other factors, such as, for example, differences in immune responses and physiological or hormonal states of the hosts (45, 46). These differences may also have a genetic basis (46, 47). However, as our results were not clear, the presence of individual variation in larval excretion should be further tested by increasing the number of samples collected for each individual. As bronchopulmonary nematodes may act as a trigger of respiratory disease (35). Further research aiming at comparing individual profiles (also assessing genetic characteristics) to parasite sensitivity/resistance could give valuable insights on the potential detrimental effects of parasites on an endangered species. Respiratory diseases are indeed a threat for the conservation of Alpine ibex and may constitute a particular problem as the species has a very low genetic variation, either in general (48), as well as at important MHC genes (7, 49, 50). Since the etiology of pneumonia, which is the main cause of mortality in the Alpine Ibex of GPNP (unpublished), is still unknown, it is of particular interest to investigate the role of pulmonary nematodes as lung lesions caused by Protostrongylids might serve as an important predisposing factor for development of bacterial pneumonia (11). Since parasitic lesions are frequently obliterated by necrosis and inflammation associated with bacterial pneumonia, the interpretation of the role of Protostrongylid parasites in the pathogenesis of pneumonia is still controversial (11, 35, 51, 52). The current study aimed at investigating individual differences in Protostrongylid prevalence and intensity. Our results showed high individual variations for the two dominant genera *Muellerius* spp. and *Protostrongylus* spp. with the latter showing also a highly aggregated distribution pattern. Despite none of the tested individual factors explained the differences in parasite infection intensity, we believe these data provide the necessary base point to further investigate how lungworm infection account for the different rates of progression of pneumonia in *C. ibex*.

## Data Availability Statement

The original contributions presented in the study are included in the article, further inquiries can be directed to the corresponding author.

## Ethics Statement

The animal study was reviewed and approved by Consiglio di Dipartimento, Scienze Veterinarie, Grugliasco, Torino, Italy.

## Author Contributions

AB, BB, EF, and SZ conceived and designed the work and drafted the manuscript and designed and carried out the process evaluation. AB, FO, EB, and SZ provided the data. All authors contributed to the analysis and interpretation of data, participated in the revision and approved the final version of the manuscript. All authors agreed to be accountable for all aspects of their respective work.

## Conflict of Interest

The authors declare that the research was conducted in the absence of any commercial or financial relationships that could be construed as a potential conflict of interest.
